# Retraction for Li et al., “The current state of research on influenza antiviral drug development: drugs in clinical trial and licensed drugs”

**DOI:** 10.1128/mbio.00106-24

**Published:** 2024-03-22

**Authors:** Yanbai Li, Shanshan Huo, Zhe Yin, Zuguang Tian, Fang Huang, Peng Liu, Yue Liu, Fei Yu

## RETRACTION

Volume 14, no. 5, e01273-23, 2023, https://doi.org/10.1128/mbio.01273-23. A number of factual errors were found in the paper. For example, the target of baloxavir marboxil was misspelled as PB1 in Table 1, and the target of favipiravir was incorrectly labeled as PA in Table 2. In addition, Paxlovid was incorrectly identified as an RdRP inhibitor. In addition to these problems, there were some spelling errors and language presentation in the article which may lead to misinterpretation of the content by readers. In order to maintain scientific rigor, we have decided to retract the article and apologize for any misinterpretation it may have caused. We apologize for any inconvenience caused by our negligence.

